# Serum Orexin A Levels and Heart Rate Variability Parameters in Newly Diagnosed, Treatment-Naïve Type 2 Diabetes Mellitus: A Case-Control Study

**DOI:** 10.7759/cureus.112979

**Published:** 2026-07-19

**Authors:** Abebe T Gebrye, Narayan Dutt Soni, Puneet Rijhwani, Aparna Garg, Md S Hussain

**Affiliations:** 1 Physiology, Mahatma Gandhi University of Medical Science and Technology, Jaipur, IND; 2 General Medicine, Mahatma Gandhi University of Medical Science and Technology, Jaipur, IND

**Keywords:** biomarker, cardiac autonomic neuropathy, heart rate variability, orexin a, treatment-naïve type 2 dm, type 2 diabetes mellitus

## Abstract

Introduction: Type 2 diabetes mellitus (T2DM) is characterized by metabolic shifts that impact neuroendocrine signals and autonomic performance, yet the direct relationship between circulating neuropeptides and cardiac regulation in early-stage disease is unclear in low-resource settings like India.

Objectives: This case-control study was performed to compare baseline serum Orexin A levels and heart rate variability (HRV) parameters between newly diagnosed, treatment-naïve T2DM patients and healthy controls, and to assess the linear correlations between serum Orexin A concentrations and HRV indices within the T2DM group.

Material and methods: One hundred newly diagnosed treatment-naïve type 2 DM and 50 age-matched healthy volunteers were recruited in the outpatient department of Endocrinology and General Medicine, Mahatma Gandhi Medical College & Hospital, Jaipur. Serum Orexin A was quantified using a competitive enzyme-linked immunosorbent assay (ELISA) kit, and five-minute resting electrocardiograms were captured to calculate time-domain parameters including the standard deviation of NN intervals (SDNN), root mean square of successive differences (RMSSD), and percentage of successive NN intervals differing by more than 50 milliseconds (pNN50) alongside frequency-domain spectral parameters including low-frequency (LF) power, high-frequency (HF) power, and the LF/HF ratio.

Result: Serum Orexin A concentrations were significantly reduced in the diabetic group compared with the healthy control (33.92 ± 28.45 pg/mL vs. 225.77 ± 20.45 pg/mL), (p < 0.001), representing a significant baseline reduction. Marked reductions were identified across all recorded autonomic components, with the mean overall time-domain tone falling below reference values (SDNN: 46.06 ± 6.16 ms vs. 85.29 ± 13.65 ms), (p < 0.001); (RMSSD: 31.50 ± 3.76 ms vs. 55.74 ± 3.23 ms), (p < 0.001). Pearson correlation revealed near-zero coefficients and no statistically significant linear association between circulating orexin A levels and any time- or frequency-domain parameter within the type 2 DM group (p > 0.05 for all).

Conclusion: In conclusion, newly diagnosed, treatment-naïve patients with T2DM exhibited significantly lower serum Orexin A concentrations and impaired HRV parameters compared with healthy controls. However, no significant linear correlations were observed between serum orexin A concentrations and HRV indices, suggesting that the relationship between circulating orexin A and cardiac autonomic function may be more complex than a simple linear association. Further studies are warranted to elucidate the underlying mechanisms.

## Introduction

Type 2 diabetes mellitus (T2DM) remains a dominant chronic metabolic disorder affecting over 463 million adults globally, with projections climbing toward 700 million by the year 2045 [1]. India contributes heavily to this global burden, serving as home to more than 77 million individuals living with diabetes [2]. Beyond the challenges of glycemic maintenance, early-stage T2DM triggers insidious systemic complications. Among these, cardiac autonomic neuropathy (CAN) is especially critical; it affects 30% to 40% of diabetic patients and significantly drives up cardiovascular mortality rates [3,4]. Heart rate variability (HRV) offers a non-invasive, highly sensitive measurement of autonomic nervous system function and is known to deteriorate during the earliest operational stages of T2DM [5-7]. A reduced overall HRV, specifically standard deviation of NN intervals (SDNN) below 50 ms, is highly associated with an elevated risk of sudden cardiac death [8,9]. However, the exact molecular signals linking early metabolic disruption to cardiac autonomic reduction remain incompletely understood.

Orexin A (hypocretin-1) is a 33-amino acid neuropeptide produced exclusively by specialized neurons within the lateral hypothalamus [10,11]. It serves as an essential central regulator of energy homeostasis, glucose utilization, sleep-wake architecture, and autonomic efferent output. Orexin A acts via two distinct G-protein-coupled receptors: OX1R and OX2R [12]. Prior clinical observations note that circulating serum orexin A levels are significantly decreased in obesity, metabolic syndrome, and long-standing T2DM [13].

Previous studies have independently examined serum Orexin A in metabolic disorders. In human study serum Orexin A in metabolic disorders were found inverse correlations between serum Orexin A and waist circumference (r = -0.60, p = 0.001) and triglycerides (r = -0.74, p = 0.001), with a positive correlation with high-density lipoprotein cholesterol (HDL) (r = 0.87, p = 0.001) in metabolic syndrome [13], and another study observed lower serum Orexin A in T2DM patients with metabolic syndrome, identifying waist circumference, triglycerides, HDL, Leptin, and tumor necrosis factor-alpha (TNF-α) as independent predictors [14]. An animal study demonstrated that administration of serum Orexin A improved glucose control and insulin sensitivity in diabetic rats [15]. Moreover, central neural mapping shows that orexinergic neurons directly innervate critical autonomic processing hubs, such as the paraventricular nucleus (PVN) and the nucleus tractus solitarius (NTS), thereby regulating downstream sympathetic and parasympathetic efferent pathways [16]. Despite these overlapping roles, no clinical human trial has yet investigated the direct association between serum orexin A levels and resting HRV parameters in newly diagnosed, entirely treatment-naïve T2DM patients. This study aims to address this gap. We hypothesized that serum orexin A levels would be significantly reduced in early T2DM and would exhibit a positive linear correlation with concurrent HRV parameters.

## Materials and methods

Study setting, design, and period

This prospective observational case-control study was conducted at Mahatma Gandhi Medical College & Hospital in Jaipur, Rajasthan, India. Study participants were recruited from both the Endocrinology and General Medicine Outpatient Departments between February 2025 and December 2025. In this study, we enrolled 150 participants who satisfied our inclusion and exclusion criteria, of which 100 were newly diagnosed T2DM patients and 50 were healthy controls according to American Diabetes Association (ADA) criteria. Prior to initiation, the institutional review board granted formal ethical clearance (reference number: MGMCAH/IEC/JPR/2023/1208, dated January 30, 2023) for the study protocol, ensuring all procedures rigidly adhered to the ethical standards outlined in the Declaration of Helsinki [17]. Every subject provided voluntary, written informed consent before participating in any screening or evaluation procedures.

Eligibility criteria

Inclusion Criteria

For T2DM group, inclusion criteria comprised age between 30 and 50 years; absence of medications known to alter heart rate variability (HRV); non-smoker status; no alcohol consumption; no substance abuse; newly diagnosed type 2 diabetes mellitus according to ADA criteria (fasting plasma glucose ≥126 mg/dL or glycated hemoglobin (HbA1c) ≥6.5%), willingness to provide written informed consent, and no personal or family history of diabetes in first-degree relatives. For the control group, inclusion criteria comprised age-matched healthy individuals aged 30-50 years, no history of diabetes (fasting blood glucose <100 mg/dL and HbA1c <5.7%), no known acute or chronic illness, and willingness to provide written informed consent were included.

Exclusion Criteria

In both groups comprised: age <30 or >50 years; established cardiovascular diseases (chronic hypertension, coronary artery disease, heart failure, or arrhythmias); other endocrine disorders (e.g., thyroid dysfunction, Cushing syndrome); chronic kidney or liver disease; neurological disorders impacting autonomic pathways; substance abuse; active prescription of medications known to alter HRV; not willing to participate and pregnancy or lactation were excluded.

Biochemical analysis and serum Orexin A measurement

A trained phlebotomist collected peripheral venous blood samples (10 mL) from the antecubital vein under aseptic conditions. All collections were performed between 9:00 AM and 11:00 AM following a 10-12 hour overnight fast. To minimize physiological variability, participants were instructed to maintain a consistent sleep schedule for three days prior to blood collection.

Routine biochemical parameters including, fasting blood glucose (FBG), glycated hemoglobin (HbA1c), liver transaminases, aspartate aminotransferase (AST) and alanine aminotransferase (ALT), and lipid fractions, total cholesterol (TC), triglycerides (TG), and high-density lipoprotein cholesterol (HDL), were processed at the MGMC&H Central Laboratory using the automated VITROS 5600 Integrated System (Ortho Clinical Diagnostics) [18]. Low-density lipoprotein cholesterol (LDL) concentrations were derived mathematically using the Friedewald equation [19].

For serum Orexin A estimation, blood samples were collected in plain serum vacutainer tubes, allowed to clot for 30 minutes at room temperature, and centrifuged at 3000 rpm for 10 minutes to separate the serum. The clear supernatant was aliquoted into sterile cryovials and stored at -80°C until analysis, with samples thawed only once to prevent degradation.

Quantitative evaluation of serum Orexin A concentrations was performed using a competitive enzyme-linked immunosorbent assay (ELISA) kit (FineTest Human OXA ELISA Kit, Catalog No. EH3487, FineTest, Wuhan, China) [20], following the manufacturer's instructions. All samples were processed in duplicate in a single batch. With intra-assay and inter-assay coefficients of variation <5.38% and <5.08%, respectively, confirming excellent precision. The kit specifically recognizes serum Orexin A with no cross-reaction with other analogs. Any sample with a duplicate coefficient of variation (CV) >10% was reanalyzed, and laboratory personnel were blinded to group allocation to eliminate measurement bias.

Assessment of autonomic cardiac tone

HRV parameters were recorded in the MGMC&H Department of Physiology Clinical Laboratory after a 10-12-hour overnight fast. HRV profiles were acquired using a PC-based, high-resolution digital ECG system (RMS Vesta 305i, RMS Medical Systems, Chennai, India) utilizing a standard sampling rate of 500 Hz. Physiological assessments were systematically scheduled from late morning to early afternoon, specifically from 09:00 AM to 13:00 PM. The evaluation environment consisted of an isolated testing laboratory maintained at a stable temperature range of 22-24°C. Each participant completed a 30-minute resting period in a supine position before a continuous five-minute raw electrocardiogram tracing was recorded for baseline data collection.

The digitized ECG signal was stored for offline analysis using the RMS Vesta 305i software. RR intervals were automatically detected by the software's built-in QRS detection algorithm. Ectopic beats were manually identified and corrected using the software's editing function. Only recordings with <5% ectopic beats were included in the final analysis. To minimize potential influence on HRV, Participants were instructed to breathe normally and spontaneously during the five-minute recording and to avoid caffeine, alcohol, and heavy meals for ≥4 hours prior to testing. Smoking and strenuous exercise were prohibited on the day of recording. All participants were asked to empty their bladder before the recording. Time-domain variables selected for evaluation included SDNN, root mean square of successive differences (RMSSD), and the percentage of successive NN intervals differing by more than 50 milliseconds (pNN50) percentage and frequency-domain components: low-frequency power (LF: 0.04-0.15 Hz) and high-frequency power (HF: 0.15-0.40 Hz), which enabled the mathematical derivation of the baseline LF/HF ratio [21].

Statistical analysis

Data analysis was performed by the Department of Community Medicine Statistician, MGMC&H, using IBM SPSS Statistics Standard Authorized User, Version 32.0 (IBM Corp., Armonk, NY, USA). Descriptive statistics for continuous clinical parameters were reported as means ± standard deviations (SD), while categorical (age and sex) variables were presented as frequencies and percentages.

Normality was assessed using the Shapiro-Wilk test, which confirmed normal distribution for all continuous variables (p > 0.05), justifying the use of parametric tests. To determine statistical variations between the T2DM group and the healthy control group, independent-samples Student's t-tests were performed.

To uncover possible linear associations between serum Orexin A concentrations and cardiac autonomic parameters, Pearson's correlation coefficient (r) was applied. Partial correlation analyses were performed adjusting for BMI, waist circumference, systolic blood pressure (SBP), and diastolic blood pressure (DBP) to account for potential confounding. For all analytical frameworks, a two-tailed alpha level set below 0.05 determined the threshold for achieving statistical significance.

## Results

Baseline, clinical, and biochemical profiles of study participants

The final analysis included 100 newly diagnosed T2DM patients and 50 healthy controls, showing age and sex (frequency matching). The case group consisted of 66 males (66.0%) and 34 females (34.0%), while the control group had 34 males (68.0%) and 16 females (32.0%). The mean age was statistically comparable between the diabetic and healthy control groups (45.63 ± 5.14 years vs. 43.90 ± 5.19 years, p = 0.056). In contrast, patients with T2DM demonstrated significantly higher BMI, waist-to-hip ratio, SBP, and DBP compared with healthy controls (all p < 0.001).

Biochemically, the T2DM group displayed marked dyslipidemia, elevated transaminases, and high glycemic indices. Peripheral serum orexin A levels were markedly reduced in T2DM patients compared with healthy controls (33.92 ± 28.45 pg/mL vs. 225.77 ± 20.45 pg/mL, p < 0.001), representing an approximately 85% lower concentration of circulating serum Orexin A, as presented in Table 1.

**Table 1:** Baseline demographic, anthropometric, metabolic, liver enzyme, serum Orexin A, characteristics of newly diagnosed patients with T2DM and healthy controls. *Statistically significant at p < 0.05. Data expressed as mean ± standard deviation. ALT: alanine transaminase; AST: aspartate transaminase; BMI: body mass index; DBP: diastolic blood pressure; FBG: fasting blood glucose; HbA1c: glycated hemoglobin; HDL: high-density lipoprotein; LDL: low-density lipoprotein; SBP: systolic blood pressure; SD: standard deviation; T2DM: type 2 diabetes mellitus. Demographics: age and gender distributions were identical between groups (p > 0.05). Metabolic shifts: T2DM patients showed significant increases in BMI, blood pressure, lipid fractions, and liver transaminases (p < 0.001). Biomarker reduction: Serum Orexin A levels were 85% lower in T2DM patients compared with healthy controls (33.92 ± 28.45 vs. 225.77 ± 20.45 pg/mL; p < 0.001).

Parameter	T2DM patients (n = 100) Mean ± SD	Healthy controls (n = 50) Mean ± SD	p-value
Demographics characteristics			
Male sex, n (%)	66 (66.0%)	34 (68.0%)	1.000
Female sex, n (%)	34 (34.0%)	16 (32.0%)	1.000
Age (years)	45.63 ± 5.14	43.90 ± 5.19	0.056
Anthropometrics and BP measurements			
Weight (kg)	74.27 ± 3.21	66.32 ± 3.84	<0.001*
BMI (kg/m^2^)	26.02 ± 0.43	23.03 ± 0.69	<0.001*
Waist-hip ratio	0.94 ± 0.02	0.78 ± 0.04	<0.001*
SBP (mmHg)	132.00 ± 2.16	119.88 ± 0.87	<0.001*
DBP (mmHg)	84.98 ± 1.61	79.52 ± 0.97	<0.001*
Metabolic and lipid profiles			
FBG (mg/dL)	216.20 ± 25.42	96.25 ± 4.80	<0.001*
HbA1c (%)	7.48 ± 0.94	5.21 ± 0.30	<0.001*
Triglycerides (mg/dL)	193.04 ± 15.64	136.19 ± 13.35	<0.001*
Total cholesterol (mg/dL)	226.56 ± 14.64	176.52 ± 12.01	<0.001*
LDL (mg/dL)	144.86 ± 30.27	134.13 ± 15.33	0.005*
HDL (mg/dL)	40.15 ± 3.90	48.30 ± 5.82	<0.001*
Liver functioning test			
ALT (U/L)	71.45 ± 5.41	50.79 ± 7.76	<0.001*
AST (U/L)	61.62 ± 6.91	41.66 ± 4.69	<0.001*
Circulating biomarker			
Serum Orexin A (pg/mL)	33.92 ± 28.45	225.77 ± 20.45	<0.001*

Autonomic performance metrics

Electrocardiographic assessment revealed significant autonomic impairment in the newly diagnosed T2DM group. Both time-domain and frequency-domain components were significantly lower in diabetic patients compared with the healthy control group (all p < 0.001). Significantly, the mean overall autonomic tone marker (SDNN) within the T2DM group was reduced to 46.06 ± 6.16 ms, which is below the 50 ms physiological level often reported as indicative of reduced HRV. Concurrently, parasympathetic markers (RMSSD and HF Power) showed significant reduction, while the LF/HF ratio, a commonly used index of sympathovagal balance, was significantly higher (1.40 ± 0.22 vs. 1.28 ± 0.15) (all p < 0.001). These parameters are presented in Table 2.

**Table 2:** Comparison of HRV parameters, time-domain, and frequency-domain parameters between newly diagnosed patients with type 2 DM and healthy controls. *Statistically significant at p < 0.05. Data expressed as mean ± standard deviation. HF: high frequency; HFnu: high-frequency normalized units; HRV: heart rate variability; LF: low frequency; LFnu: low-frequency normalized units; LF/HF: low-frequency to high-frequency ratio; NN50: number of pairs of successive NN intervals that differ by more than 50 ms; pNN50: percentage of successive NN intervals differing by more than 50 ms; RMSSD: root mean square of successive differences; SD: standard deviation; SDNN: standard deviation of NN intervals; T2DM: type 2 diabetes mellitus. Autonomic reduction: All time- and frequency-domain HRV metrics were significantly reduced in the T2DM group (p < 0.001). Pathologic threshold: Mean SDNN in diabetic patients decreased to 46.06 ± 6.16 ms, falling below the statistical threshold of 50 ms. Sympathovagal shift: reduced RMSSD and a higher LF/HF ratio (1.40 ± 0.22 vs. 1.28 ± 0.15) confirm significant parasympathetic withdrawal and relative sympathetic dominance.

HRV parameter	T2DM (n = 100) Mean ± SD	Healthy controls (n = 50) Mean ± SD	p-value
Time-domain parameters			
SDNN (ms)	46.06 ± 6.16	85.29 ± 13.65	<0.001*
RMSSD (ms)	31.50 ± 3.76	55.74 ± 3.23	<0.001*
NN50 (count)	12.47 ± 4.07	15.10 ± 2.19	<0.001*
pNN50 (%)	11.53 ± 2.05	16.78 ± 3.35	<0.001*
Frequency-domain parameters			
LF power (ms²)	327.52 ± 27.82	885.16 ± 122.02	<0.001*
HF power (ms²)	235.75 ± 27.71	685.26 ± 111.70	<0.001*
Total power (ms²)	561.92 ± 41.41	1571.58 ± 222.14	<0.001*
LF (nu)	58.27 ± 3.43	56.37 ± 2.20	<0.001*
HF (nu)	41.69 ± 3.40	43.62 ± 2.20	<0.001*
LF/HF ratio	1.40 ± 0.22	1.28 ± 0.15	<0.001*

Biomarker-autonomic correlation framework

Despite the significant reduction in both circulating serum orexin A levels and autonomic performance markers within the T2DM group, Pearson correlation demonstrated that no statistically significant linear associations were observed. Serum orexin A content demonstrated no statistically significant correlation with any time-domain or frequency-domain HRV metric. The individual correlation statistics for the diabetic group are detailed in Table 3.

**Table 3:** Pearson correlation between serum Orexin A concentration and HRV parameters among newly diagnosed T2DM patients (n=100). Note: all p-values >0.05, demonstrating non-significant linear correlation. HF: high frequency; HFnu: high-frequency normalized units; HRV: heart rate variability; LF: low frequency; LFnu: low-frequency normalized units; LF/HF: low-frequency to high-frequency ratio; ms: milliseconds; ms²: square milliseconds; NN50: number of pairs of successive NN intervals that differ by more than 50 ms; p-value: statistical probability value; pNN50: percentage of successive NN intervals differing by more than 50 ms; r-value: Pearson correlation coefficient; RMSSD: root mean square of successive differences; SDNN: standard deviation of NN intervals; T2DM: type 2 diabetes mellitus. No linear link: Pearson correlation shows a near-zero association between Serum Orexin A and all HRV parameters. Statistical significance: All calculated correlation values yielded non-significant results (p > 0.05 for all parameters). Clinical conclusion: Peripheral serum Orexin A reduction and early cardiac autonomic function may be more complex than a simple linear association. Further studies are warranted to elucidate the underlying mechanisms in early-stage diabetes.

HRV parameter	Mean ± SD	r- value	p-value
SDNN (ms)	46.06 ± 6.16	-0.008	0.938
RMSSD (ms)	31.50 ± 3.76	-0.084	0.404
NN50 (count)	12.47 ± 4.07	0.006	0.951
pNN50 (%)	11.53 ± 2.05	-0.149	0.140
LF power (ms^2^)	327.52 ± 27.82	-0.116	0.250
HF power (ms^2^)	235.75 ± 27.71	0.045	0.660
Total power (ms^2^)	561.92 ± 41.41	-0.048	0.635
LF (nu)	58.27 ± 3.43	-0.111	0.272
HF(nu)	41.69 ± 3.40	0.106	0.295
LF/HF ratio	1.40 ± 0.22	-0.094	0.351

## Discussion

To the best of our knowledge, this study is among the first to evaluate the direct relationship between peripheral serum orexin A concentrations and resting HRV indices in newly diagnosed, treatment-naïve T2DM patients. Our investigation produced three primary findings. First, serum orexin A levels are significantly lower in early T2DM patients (33.92 ± 28.45 pg/mL; p < 0.001), relative to controls (225.77 ± 20.45 pg/mL; p < 0.001), confirming a substantial approximately 85% systemic reduction. This outcome aligns with observations by Rani et al. [14], who identified comparable concentrations of 25-35 pg/mL in patients with long-standing diabetes and concurrent metabolic syndrome. Previous studies by Gupta et al. [13] established that orexin A levels are reduced under conditions of obesity and metabolic syndrome. Our data extend these findings by isolating this significant reduction directly at the time of clinical diabetes diagnosis, explicitly removing the confounding effects of using medications or lifestyle modifications.

Second, we observed significant and widespread cardiac autonomic dysfunction in these newly diagnosed individuals. Time- and frequency-domain HRV parameters were significantly reduced. The mean SDNN (46.06 ± 6.16 ms) fell below the threshold level of 50 ms, which is clinically associated with an increased vulnerability to sudden cardiac death [8]. Concurrently, the significant reductions in RMSSD (31.50 ± 3.76) and HF power (235.75 ± 27.71) suggest reduced parasympathetic activity, which has been reported as an early feature of diabetic CAN [4]. These findings expand on previous cross-sectional literature [5-7] by demonstrating that substantial cardiac autonomic impairment is already established at the clinical onset of diabetes.

Thirdly, despite the significant reductions in both orexin A and HRV parameters, we found no statistically significant linear correlation between peripheral orexin A levels and any autonomic parameter. This finding adds to the limited available evidence and suggests that serum orexin A levels were not linearly associated with HRV parameters in early T2DM, which may show a more complex association than a simple linear one.

Several confounding factors warrant consideration. The T2DM group had significantly higher BMI, waist circumference, blood pressure, and adverse lipid profiles compared to controls. These variables independently affect both Orexin A levels and HRV measures. Obesity and adiposity are associated with reduced circulating orexin A levels [13,14]. Similarly, elevated blood pressure and dyslipidemia impair autonomic function [5,7]. To address these potential confounders, we performed partial correlation analyses adjusting for BMI, waist circumference, systolic blood pressure, and diastolic blood pressure. The non-significant correlation persisted after adjustments, suggesting a non-significant linear association is not merely an artifact of group differences. However, residual confounding from unmeasured variables cannot be excluded.

Our findings of no significant linear correlation contrast with animal studies demonstrating direct autonomic effects of central orexin A administration. Animal study demonstrated that orexin A microinjection into the rat paraventricular nucleus increased sympathetic nerve activity [22]. However, these animal studies utilized high pharmacological doses delivered directly into the cerebral ventricles, whereas our study tracked endogenous peripheral levels in humans. Animal studies are experimental and context-specific, and their findings may not directly translate to human physiology.

The absence of a linear correlation in our study may stem from the physiological divergence between peripheral serum Orexin A and central hypothalamic orexinergic activity. Serum Orexin A is produced primarily by neurons in the lateral hypothalamus, functioning as a neurotransmitter regulating autonomic and metabolic pathways [10,11], via orexinergic neurons that project to autonomic processing centers such as the PVN and NTS [16]. Peripheral blood concentrations may not fully mirror central hypothalamic orexinergic tone due to separate clearance rates, systemic carrier proteins, and minor peripheral production sites. Thus, serum measurement may not capture central hypothalamic efferent activity directly responsible for downstream cardiac modulation. The lack of correlation observed in the study findings should be interpreted within the context of the study population: newly diagnosed, treatment-naïve T2DM patients from a single tertiary care center in Jaipur, Rajasthan, India.

Clinical implications and study limitations

Several limitations of this study require acknowledgment. First, the observational, cross-sectional design prevents determination of causal pathways. Second, short-term (five-minute) HRV recordings do not capture circadian variations in autonomic function. Thirdly, triglyceride measurements are highly variable and may not reflect long-term lipid status. Fourth, this was a single-center study, so our findings may not generalize to other populations. Fifth, we did not measure small, dense LDL particles, which may be more relevant to cardiovascular risk than total LDL. Sixth, we did not assess inflammatory markers (e.g., high-sensitivity C-reactive protein (hs-CRP), IL-6) that could mediate the lipid-HRV relationship. Seventh, we did not evaluate dietary patterns or physical activity levels, which could confound the observed associations.

Despite limitations, our study has several clinical implications. Peripheral orexin A may serve as a sensitive biomarker for early metabolic dysfunction. Comprehensive HRV screening should be initiated at T2DM diagnosis, as our study already demonstrated SDNN values below 50 ms [8]. While therapeutic interventions like physical exercise or weight reduction may help restore circulating Orexin A levels, their effects on cardiac autonomic protection cannot be assumed based on serum Orexin A changes alone and must be tracked independently. The lack of correlation suggests these biomarkers capture distinct aspects of pathophysiology and should be considered complementary. The prognostic value of serum Orexin A and HRV for predicting cardiovascular outcomes remains to be established in prospective longitudinal studies.

## Conclusions

In conclusion, newly diagnosed, treatment-naïve patients with T2DM exhibited significantly lower serum orexin A concentrations and impaired HRV parameters compared with healthy controls. However, no significant linear correlations were observed between serum orexin A concentrations and HRV indices in this study. This suggests that the relationship between circulating orexin A and cardiac autonomic function in early T2DM may be more complex than a simple linear association. Some explanations for this lack of correlation are possible, including nonlinear associations, threshold effects, measurement variability, or the possibility that peripheral serum orexin A does not fully capture central orexinergic activity. Future longitudinal and mechanistic studies are warranted to elucidate the underlying relationships between orexinergic signaling and autonomic function in T2DM.

## References

[1] Saeedi P, Petersohn I, Salpea P (2019). Global and regional diabetes prevalence estimates for 2019 and projections for 2030 and 2045: results from the International Diabetes Federation Diabetes Atlas, 9(th) edition. Diabetes Res Clin Pract.

[2] Pradeepa R, Mohan V (2021). Epidemiology of type 2 diabetes in India. Indian J Ophthalmol.

[3] Valensi P, Pariès J, Attali JR (2003). Cardiac autonomic neuropathy in diabetic patients: influence of diabetes duration, obesity, and microangiopathic complications—the French multicenter study. Metabolism.

[4] Vinik AI, Maser RE, Mitchell BD, Freeman R (2003). Diabetic autonomic neuropathy. Diabetes Care.

[5] Coopmans C, Zhou TL, Henry RM (2020). Both prediabetes and type 2 diabetes are associated with lower heart rate variability: the Maastricht study. Diabetes Care.

[6] Kudat H, Akkaya V, Sozen AB (2006). Heart rate variability in diabetes patients. J Int Med Res.

[7] Voroneanu L, Nistor I, Dumea R, Apetrii M, Covic A (2016). Silymarin in type 2 diabetes mellitus: a systematic review and meta‐analysis of randomized controlled trials. J Diabetes Res.

[8] Malik M, Bigger JT, Camm AJ (1996). Heart rate variability: standards of measurement, physiological interpretation, and clinical use. Eur Heart J.

[9] Kleiger RE, Miller JP, Bigger JT (1987). Decreased heart rate variability and its association with increased mortality after acute myocardial infarction. Am J Cardiol.

[10] Sakurai T, Amemiya A, Ishii M (1998). Orexins and orexin receptors: a family of hypothalamic neuropeptides and G protein-coupled receptors that regulate feeding behavior. Cell.

[11] de Lecea L, Kilduff TS, Peyron C (1998). The hypocretins: hypothalamus-specific peptides with neuroexcitatory activity. Proc Natl Acad Sci U S A.

[12] Scammell TE, Winrow CJ (2011). Orexin receptors: pharmacology and therapeutic opportunities. Annu Rev Pharmacol Toxicol.

[13] Gupta V, Mishra S, Kumar S (2015). Association of circulating orexin-A level with metabolic risk factors in North Indian pre-menopausal women. Indian J Physiol Pharmacol.

[14] Rani M, Kumar R, Krishan P (2019). Serum level of orexin A and its correlation with metabolic risk factors in type 2 diabetes mellitus patients. Int J Diabetes Dev Ctries.

[15] Kaczmarek P, Skrzypski M, Pruszynska-Oszmalek E (2017). Chronic orexin-A (hypocretin-1) treatment of type 2 diabetic rats improves glucose control and beta-cell functions. J Physiol Pharmacol.

[16] Peyron C, Tighe DK, van den Pol AN, de Lecea L, Heller HC, Sutcliffe JG, Kilduff TS (1998). Neurons containing hypocretin (orexin) project to multiple neuronal systems. J Neurosci.

[17] World Medical Association (2013). World Medical Association Declaration of Helsinki: ethical principles for medical research involving human subjects. JAMA.

[18] Ortho-Clinical Diagnostics, Inc. Inc. (2015). VITROS Chemistry Products HbA1c Reagent Kit, VITROS Chemistry Products Calibrator Kit 31, VITROS Chemistry Products %A1c Performance Verifiers I and II. U.S. Food and Drug Administration.

[19] Friedewald WT, Levy RI, Fredrickson DS (1972). Estimation of the concentration of low-density lipoprotein cholesterol in plasma, without use of the preparative ultracentrifuge. Clin Chem.

[20] Fine Biotech Kit (2019). Instructions for Use: Human Orexin A (OXA) Competitive ELISA Kit. https://www.fn-test.com/product/eh3487/.

[21] Manzella D, Paolisso G (2005). Cardiac autonomic activity and type II diabetes mellitus. Clin Sci (Lond).

[22] Fan Y, Jiang E, Hahka T, Chen QH, Yan J, Shan Z (2018). Orexin A increases sympathetic nerve activity through promoting expression of proinflammatory cytokines in Sprague Dawley rats. Acta Physiol (Oxf).

